# *“Then they prayed*, *they did nothing else*, *they just prayed for the boy and he was well”*: A qualitative investigation into the perceptions and behaviours surrounding snakebite and its management in rural communities of Kitui county, Kenya

**DOI:** 10.1371/journal.pntd.0010579

**Published:** 2022-07-06

**Authors:** Leo Wood, Cecilia Ngari, Stanley Parkurito, Kieran Barnes, Denis Otundo, Daniel Asiago Misiani, Geoffrey Maranga Kephah, Anna Trelfa, George O. Olouch, Robert A. Harrison, Frank-Leonel Tianyi

**Affiliations:** 1 Centre for Snakebite Research & Interventions, Liverpool School of Tropical Medicine, Pembroke Place, Liverpool, United Kingdom; 2 Kenya Snakebite Research & Intervention Centre, Institute of Primate Research, Karen, Nairobi, Kenya; 3 Centre for Neglected Tropical Diseases, Liverpool School of Tropical Medicine, Pembroke Place, Liverpool, United Kingdom; Sir Salimullah Medical College, BANGLADESH

## Abstract

**Introduction:**

Human-snake interactions are common in tropical regions where subsistence-farming and livestock-herding activities predominate alongside proliferation of snakes. Local beliefs and perceptions about snakes and snakebites influence human behaviour. Understanding these beliefs and perceptions can inform the development of resources to drive behaviour change and to minimise the risk of injury to both humans and snakes. This qualitative study, conducted between May and July 2019, sought to explore the beliefs and perceptions regarding snakes and snakebites, and methods of prevention and management among members of the community in Kitui County, Kenya.

**Methods:**

Semi-structured interviews were used to collect qualitative data from 23 participants, recruited using a stratified purposeful sampling strategy in four selected sub-counties of Kitui county. Interview data was anonymised and coded and a thematic analysis was conducted using NVivo 12.

**Results:**

People from Kitui county mostly had negative perceptions about snakes. There was a generalised awareness of the need to prevent snakebite, predominantly through keeping snakes away from homes/compounds. However, implementation was limited by financial constraints. Participants also identified logistic and financial obstacles to early hospital presentation following a snakebite, and they expressed a strong preference of having their snakebites treated in a hospital over consulting traditional healers. There was a universal recognition of the benefit of early intervention with a specific appreciation of the utility of the black stone. Furthermore, the removal of a snake’s “teeth” was an expected treatment outcome for some community members, with the failure to do so perceived as causing poor wound healing or persistence of symptoms. Some religious groups held views which differed from most participants.

**Conclusion:**

There is a need to explore and clarify common misconceptions about snakes and first aid treatment of snakebites, encourage learning about the true nature of snakes, and highlight beneficial uses of snakes. A change in the epistemological conception of community education material by enhancing the value and use of local forms of knowledge, and the employment of art techniques to transmit this knowledge, could improve community perception and methods of snakebite prevention. Patient expectations should be appropriately managed by discussing possible outcomes, incorporating follow-up visits and addressing long-term complications of snakebites.

## Author summary

Snakebites mostly affect rural communities living in tropical regions. The beliefs and perceptions of these rural inhabitants on snakes and snakebites could inform the development of resources to minimise the risk of injury to humans and snakes. However, few studies have investigated how rural communities perceive snakes and snakebites, and the beliefs that drive these perceptions. We conducted interviews with members of the community in Kitui county, to explore the beliefs and perceptions regarding snakes and snakebites and methods of prevention and management. Negative perceptions were common, and there was a generalised awareness to prevent snakebite by keeping snakes away from homes/compounds. There was a preference for hospital treatment of snakebite, but this was limited by logistic and financial obstacles to early hospital presentation. Most inhabitants used the black stone before seeking health care, and there was an expectation for the snake’s ‘teeth’ to fall out of the wound for complete healing to occur. Our findings highlight a need to explore and clarify common misconceptions about snakes and first aid treatment of snakebites, and to improve community perceptions using locally developed forms of knowledge, and employment of art techniques such as music and dance.

## 1.0 Introduction

Snakebite is a disease of tropical poverty which mostly affects poor rural farmers in tropical regions [1]. Favourable geographic and environmental conditions provide supportive habitats for a variety of snakes. Amongst these are venomous snakes which can cause life threatening envenoming or lifelong disability [2,3]. Human-snake interactions are amongst the most common human-wildlife confrontations, especially in Sub-Saharan Africa where subsistence farming dominates agricultural activity, where houses are located close to natural snake habitats, where the use of inexpensive and locally available building materials result in homes with little protection from snakes, and where communities are located several hours from effective healthcare [1,4,5]. While some interactions are inevitable, local beliefs and customs about snakes, snakebite and its treatment influence how people behave when they see a snake and when they are bitten [6,7]. Understanding these beliefs and perceptions can inform development of resources to drive behaviour change towards snakes and minimise the risk of injury to both humans and snakes following a human-snake interaction.

There are 140 known snake species in Kenya, of which 29 are venomous and 13 are medically important. Envenoming by spitting cobras, puff adders, neurotoxic cobras and mambas are reported to dominate snakebite incidences [8]. Reducing the burden of snakebites requires preventing them from occurring in the first place and managing them effectively when they do occur. Previous studies have revealed a delay in seeking hospital treatment following a snakebite, with patients using traditional early intervention measures such as tourniquets, incision at the bite site, ingestion of medicinal herbs or application of the black stone [9,10]. Some victims use these remedies at home while others prefer to consult traditional healers with experience in assisting snakebite victims. In the absence of any medical benefit of these techniques, the pervasive nature of their use requires a deeper understanding of the local beliefs and perceptions that drive these attitudes [11].

In 2019, the WHO released its strategy to halve the morbidity and mortality from snakebite envenoming by 2030 [12]. Empowering and engaging communities to prevent snakebite is one of the main pillars of the strategy, signalling a need to adopt a ‘biosocial approach’ for a better understanding of the social, economic and political contexts within which snakebites occur and a call for more social research [13,14]. Qualitative research permits an exploration and understanding of the role that snakes play in the lives of rural community members, the meaning they attribute to snakes and snakebite and the actions they take to prevent and manage snakebites [15]. This qualitative study sought to explore the beliefs and perceptions regarding snakes and snakebites and methods of prevention and management among members of communities in four sub-counties in Kitui County, Kenya.

## 2.0 Methods

### 2.1 Ethics statement

Ethical approval for the study was obtained from the Kenyatta National Hospital–University of Nairobi Ethics Research Council, Nairobi, Kenya (P348/05/2019) and the Liverpool School of Tropical Medicine MSc Review Panel (Application 1908). Written informed consent was obtained from participants prior to starting the interview. Pseudonymised data was used for the analysis and patient anonymity was maintained throughout. No compensation was offered to interviewees.

### 2.2 Study site

**Fig 1:**
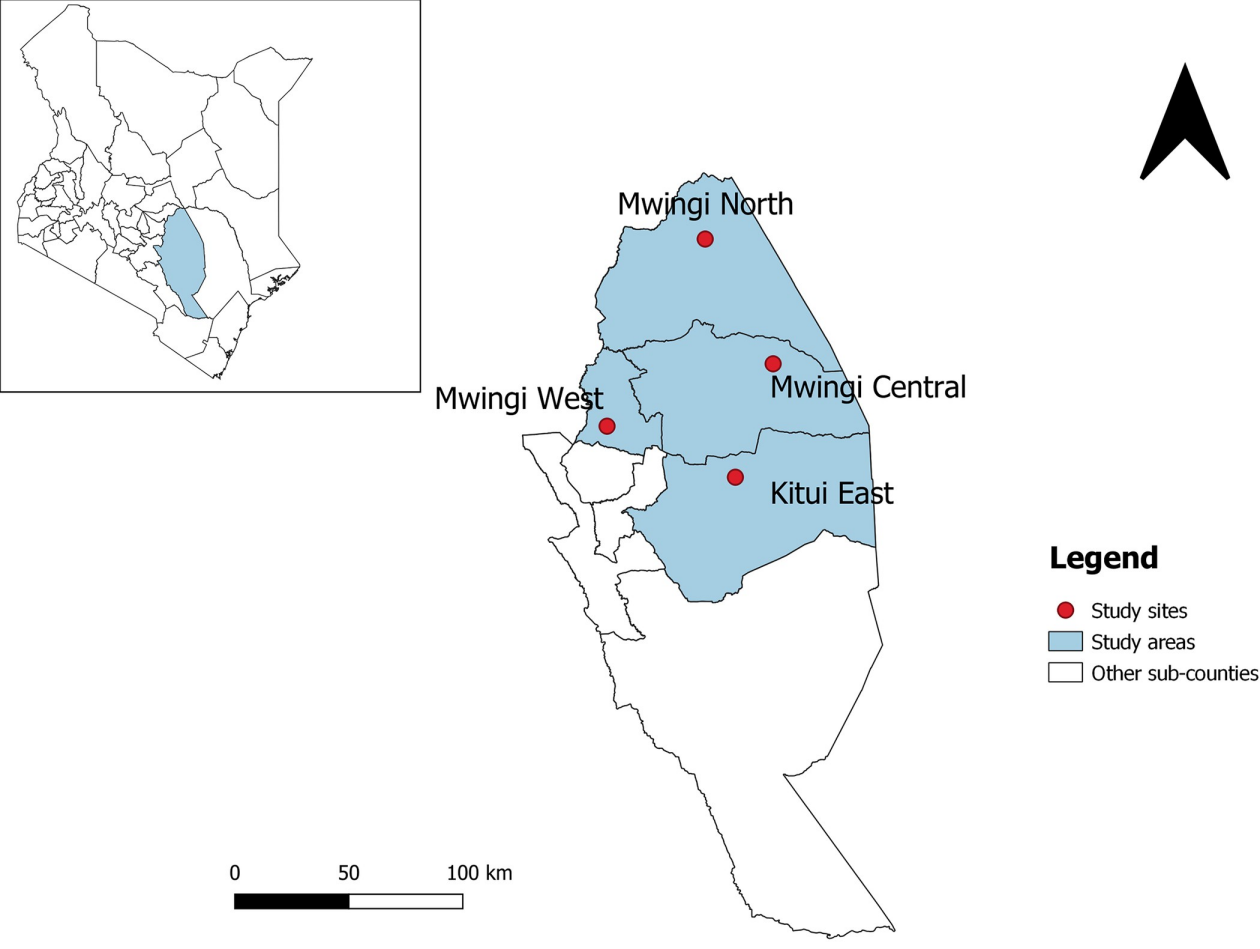
Map of Kitui county showing the study areas and sites *(Created using the Free and Open Source QGIS v3*.*22*.*3)*.

Kitui county is located in the east-central part of Kenya, about 160km from Nairobi. It covers an area of approximately 30,496 km2 with a projected population of 1,123,401 inhabitants in 2018. The county has an arid/semi-arid climate with increasingly unpredictable rainfall patterns. Over 85% of the county resides in rural areas, practicing subsistence agriculture and keeping livestock. Poverty levels are high (47.5%) and only 4.8% of the county has access to electricity [16].Climatic and environmental conditions favour the presence of a variety of venomous and non-venomous snakes, of which the most medically important are considered to be; red spitting cobra (*Naja pallida*), black-necked spitting cobra (*Naja nigricollis*), puff adder (*Bitis arietans*), neurotoxic cobras (*Naja haje* and *Naja melanoleuca*) and black mamba (*Dendroaspis polylepis*) [8]. This study was conducted in four sub-counties with varying population densities and snakebite incidence: Mwingi North, Mwingi central, Mwingi west and Kitui east (Fig 1). This study was one of several studies undertaken by the African Snakebite Research Group (ASRG) and involved the Kenya Snakebite Research and Intervention Centre (KSRIC) and the Liverpool School of Tropical Medicine (LSTM).

### 2.3 Study design

A qualitative research design was used to explore the beliefs and perceptions of community members regarding snakes and snakebites, and to assess their attitudes towards the prevention and management of snakebites [17]. We used semi-structured interviews to illicit participants’ feelings, thoughts and experiences of snakes or snakebite and to allow them to explore traumatic or sensitive topics [15].

### 2.4 Sampling and recruitment

Kitui County is divided into eight sub-counties which are further subdivided into 40 wards and 247 county villages. The village is the lowest level of the county administrative unit. We used a stratified purposeful sampling, which is a ‘statistically non representative stratified sampling’ technique [18], enabling us to recruit participants in each of the four selected sub-counties. Villages were selected based on accessibility and familiarity to the community health volunteers, and households were randomly selected from each village using a random number method. Table 1 shows the selected villages and the number of participants selected from each village. Data saturation was achieved after recruiting 23 participants with the non-emergence of new themes [19].

**Table 1:** Selected villages and number of participants recruited in each village.

Sub-county	Ward	Location	Number of interviews
Mwingi Central	Nguni	Nguni	4
		Imba	2
Mwingi North	Kyuso	Ngaai	4
		Kimangaoui	2
Mwingi West	Nguutani	Nzawa/ Kakululo	3
	Kiomo/ Kethyani	Kiomo	2
Kitui East	Mutitu/ Kaliku	Mutitu	6
**Total**			**23**

### 2.5 Data collection (May–July 2019)

An initial topic guide was developed from the key themes identified in the literature regarding the beliefs and perceptions of snakes and snakebites, and the attitudes towards snakebite prevention and management. This was modified following discussions with snakebite experts, local researchers, and public health authorities. The guide was pre-tested, piloted and adapted to suit cultural specificities prevailing in Kitui. Further iterative revisions were made throughout the data collection process with the incorporation of new experiences as they arose [20].

Data was collected by the first author (LW) between May and July 2019 with the help of an interpreter who was familiar with the local language and culture of the area. LW carried out this study as part of his MSc project at LSTM and he received training in qualitative research methods. The interpreter received training in interpreting for qualitative interviewing and participated in the pilot of the topic guide. A copy of the final topic guide can be found in S1 Table.

Interviews were conducted in the Kikamba language, in an area of the interviewee’s choosing, mostly in an open space close to their houses. Interviews lasted between 30 minutes to an hour, at a time that suited the participants [21].

### 2.6 Data analysis

The interviews were recorded and transcribed verbatim using the *f4transkript* software package. Transcripts were anonymised and coded using NVivo 12.0 software. The first author repeatedly read the transcripts and developed a coding framework for analysis. Two other authors independently coded a subset of the data and further discussed the coding framework in a collaborative and reflexive manner. A thematic analysis approach was used, and a framework was iteratively developed upon the emergence of new themes and individual codes were grouped into categories until a finalised framework was achieved. The codes were iteratively compared within and across transcripts, and the coded extracts were summarised and grouped into themes and sub-themes as appropriate [22].

### 2.7 Reflexivity

The main data collector was a white male British student who did not know the local language. His mere presence may have influenced the responses of the participants, for example, when describing a snakebite incident, one participant said the snake had the same colour as the skin of the first author, and that this was a friendly snake (even though she had been bitten by the snake). He was conscious of the influence of his background and positionality on the conduct and interpretation of the data. Keeping a reflective log and reassuring participants of the confidentiality of their responses helped mitigate the ‘interviewer’ effect [23,24].

## 3.0 Results

A total of 23 interviews were conducted with an almost equal distribution across the four sub-counties. Most of the participants were in the 31–50 years age group and about a third of them had personally experienced a snakebite. Sociodemographic characteristics of participants are summarised in Table 2.

**Table 2:** Sociodemographic characteristics of study participants.

Characteristics	Total N = 23
Gender	
Male	12
Female	11
Age categories	
< = 30	5
31–50	10
>50	8
Occupation	
Home keeper	5
Farmer	4
Pastoralist	6
Professional	3
Unemployed/other	5
Snakebite encounter	
Self	7
Other	11
None	5
Attended a Barraza (Community education session organised by K-SRIC)	
Yes	6
No	17

### 3.1 Perceptions and beliefs about snakes and snakebites

#### 3.1.1 Perceptions and beliefs abouts snakes

*3*.*1*.*1*.*1 They are harmful and dangerous animals and should be killed especially when they enter the house*. There was a unanimous negative perception of snakes with participants attributing names ranging from “the enemy”, “dangerous animals” to “Satan”. All participants thought that some snakes were more poisonous than others, but a few felt that all snakes were harmful. Most participants felt that snakes should be killed on sight, especially if they entered the house or the compound. Some participants insisted on burning the dead snake after it had been killed. This sentiment was strongest among pastoralists who kept chickens or other livestock, with one participant willing to dig a hole to kill a snake. Participants who had previously been bitten were much more scared of snakes compared to the rest.

“*Most of the time he tries to kill them*, *even if they enter in the holes*. *He digs for them so that he can kill them*” (Int 12)

“…*the bones of the dead snake*, *they are very poisonous so that is why they have to burn them…if you step on those bones*, *it is the same as being bitten by that snake*” (Int 10)

“*She was always scared of them [snakes]*, *but now she fears them so much*, *even now*, *if she sees a lizard*, *she runs away fearing it is a snake*” (Int 13)

*3*.*1*.*1*.*2 They are everywhere*. All participants agreed on the ubiquitous nature of snakes. They saw snakes in their homes, their compounds, fences and along pathways. They agreed that snakes were more common in the forests than in their immediate surroundings, and that snakes ventured into their homes in search of food or water.

“…*He is saying they [snakes] are many in this area*. *So*, *he sees them every day*, *even you can see many snakes per day*. *So*, *he says*, *even if you cannot see the snake*, *you can see its trail*, *when it is crossing the road*.” (Int 12)

*3*.*1*.*1*.*3 They run away when you see them*. Some participants described snakes as “fearing” humans and they said most snakes would try to move away whenever they meet a human. These participants agreed that they would not attempt to kill a snake if they saw it in a forest and that they felt some snakes were harmless.

“*Even those snakes*, *there are some snakes that see you and they run away before you decide to kill it*.” (Int 3)

#### 3.1.2 Perceptions about snakebites

Participants were split in their beliefs on why people are bitten by snakes. About half of the participants attributed a snakebite to an accidental occurrence with some participants comparing it to a road traffic accident.

“…*they only bite people by mistake*, *as an accident*, *like the way people die on the road*…” (Int 3)

A smaller group of participants claimed snakes were destined to bite humans, citing religious texts to support their opinions.

“*The Bible says that from the beginning the snake was to bite people*, *so he believes that is the only reason as to why people are bitten*, *from what happened in the garden of Eden*” (Int 22)

A final group viewed a snakebite as an intentional act attributed to witchcraft and sorcery. They felt the witch doctors sent snakes to bite people out of retribution or jealousy, and that bites from such snakes were always fatal.

“*Sometimes witch doctors might send a snake when they see you are proceeding well in life*. *They can send a snake to you and then it bites you or your child…If you are bitten by the snake that is sent*, *even if you are taken to the hospital*, *even if you are treated you cannot recover and you die*” (Int 13)

### 3.2 Knowledge and attitude about preventing snakebites

#### 3.2.1 Keeping snakes away from homesteads

All participants mentioned at least one method of keeping snakes away from homes/compound. Interventions included using durable building materials (concrete, solid roofing material, etc), keeping the surroundings clean and clear, burning of trees, burning of rubber, and spraying of paraffin around the house.

“… *only cleaning the compound and keeping it clear…snakes do not like places that are clear and that is why most of the time they spend there in the forest*…” (Int 10)

“*There is a type of tree*, *it is called a matanga*, *that one they use*, *they burn it*, *and the smell keeps the snake away or they burn the rubber shoe*” (Int 8)

“…*uses the sole of these shoes*, *the ones that are not being used*, *she burns them*, *and she even uses paraffin*, *she pours around the house so that the snakes do not come*…” (Int 21)

#### 3.2.2 Preventing snakebites

Most participants knew the recommended practices to prevent snakebites such as wearing closed shoes and using torches at night. They however did not fully apply this knowledge due to financial limitations or unfavourable climatic conditions. Two participants felt that only prayers could prevent a snake from biting themselves or their loved ones.

“*When there is rain and they are cultivating … they usually wear closed shoes to avoid being bitten by snakes*, *but they have no money to buy gum boots*.” (Int 3)

“…*only prayers*. *Only prayers can protect him from being bitten*” (Int 23)

### 3.3 Knowledge and attitudes on the management of snakebites

#### 3.3.1 Health seeking behaviour

Most participants agreed on the need to attend a hospital following a snakebite. Only a handful of them said they would visit a traditional healer before going to a health facility. Most of the participants seemed offended at the question about visiting a traditional healer, with some citing religious reasons or poor mastery of the way traditional medicine works as the main reasons for not visiting a traditional healer. There were some participants who believed in prayers as the sole treatment for snakebites. One participant previously visited health facilities, but now only believed in prayers, and he mentioned a few biblical verses in support of his decision.

“… *does not know how they [traditional healers] treat people or how they work so she does not go there*.” (Int 8)

“… *he is a Christian and so they do not go there because they are saved* …” (Int 10)

“…*where he was bitten it had changed colour and become very black and where he was bitten was very painful*, *but after they said prayers he felt very well again and he recovered … Prayer is the only thing… James chapter 5 verse 13*. *The topic is powers of prayers*…” (Int 23)

#### 3.3.2 Perceived challenges in seeking health care

*3*.*3*.*2*.*1 Poor road networks and inappropriate transport*. All participants who decided to go to a hospital expressed difficulties in reaching health facilities. The major challenges were bad roads and lack of a readily available ambulance. Most of the participants used a motorbike, some used a bicycle, while a few had to walk to the health facility. Participants admitted to spending significant sums of money on transportation fares and some participants identified these expenditures as a deterrent to seeking hospital care.

“… *due to the lack of proper roads*, *they used motorbikes*…” (Int 7)

“… *there were no motorbikes*, *so they used a bicycle*. *He went on the back*…” (Int 10)

*3*.*3*.*2*.*2 Financial restrictions in accessing healthcare*. Some participants mentioned financial restrictions as a reason for delaying hospital presentation. The need to have a minimum amount of money before going to a hospital and the fear of being detained in hospital until the bills had been settled. These and related financial considerations influenced the speed with which some victims arrived at a hospital or whether they decided to go at all. One participant recognised that traditional doctors were sometimes preferred because they would accept payments at a later date.

“…*when she does not have money*, *that is a reason she would not go immediately*. *Because if you take the person there to the witch doctor*, *you can go and talk to her*, *then she can remove those teeth and you can come back and search for the money to pay her*. *Whereas*, *when you go to the hospital you cannot leave until you have paid*.” (Int 3)

#### 3.3.3 Early intervention (“First aid”) methods used in Kitui county

All participants knew at least one early intervention method for use after a snakebite, including applying/ingesting a black stone, use of tourniquets, ingesting uncooked eggs and using topical herbs. Black stone was the most common early intervention method and the usage differed among the participants. Some participants placed it over the bite wound, others crushed the stone and drank it mixed with water, while some boiled the black stone with sugar and water before placing it over the bite wound. Most participants sought black stone before leaving for the hospital and they left the stone in place while receiving treatment at the hospital. There was no obvious difference in knowledge or proclivity to use early intervention among participants who attended or did not attend community engagement activities organised by K-SRIC.

“… *they smashed it into a dust*, *then you mix it with water*, *and you give it to the patient to drink*. *First of all*, *they did that and then they took him to the hospital … you drink so that you can vomit the poison … he vomited when he got to the hospital*…” (Int 8)

“… *they just boil it with sugar in water and then they take it out and leave it for a while to cool*. *Then you apply where he or she has been bitten…they cut to make it bleed* …” (Int 8)

“… *when you are bitten by a snake they used eggs so that*, *if you take that egg*, *uncooked egg*, *so that you take it and you vomit the poison out*.” (Int 1)

“… *when he was bitten*, *he put that*, *fodda*… *it is like a red powder*, *you put where you have been bitten* …” (Int 1)

#### 3.3.4 Falling of the teeth as the endpoint of treatment

Some participants associated complete healing with the removal of “teeth” (fangs) or their falling off from the bite wound. They interpreted the black stones falling off the wounds as the “teeth” falling off. They also associated poor wound healing or persistence of symptoms in the affected body part with incomplete removal of the fangs. This version of healing was most common among participants who had personally experienced a snakebite.

“… *he had been given some tablets*, *but the problem was that they had not removed the teeth … he was taking the tablets now he was at home*, *the finger was still painful … the teeth had interfered with the muscles of the finger*. *So*, *the hand was swollen*. *So he went to the hospital*, *that is when they removed the teeth*. “(Int 15)

“… *he took the baby there so that they can confirm whether there are no teeth left in and that the baby is ok*.*”* … (Int 15)

“… *he was injected with some medications and the black stone*, *it took around 12 hours to fall off*. *And when it fell off*, *the teeth came out with the stone*. “(Int 10)

## 4.0 Discussion

### 4.1 Key findings

These rural villagers in Kitui county mostly had negative perceptions about snakes. They felt that snakes were dangerous and harmful animals, sometimes sent by Satan or witch doctors, and that they deserved to be killed on sight. They frequently encountered snakes in their surroundings, and some felt that snakes feared humans and would run away if given the chance. Community perceptions of snakebite varied between unlucky bites following a human-snake interaction, intentional bites by snakes sent by malicious supernatural beings, and predetermined bites prescribed in biblical texts by God. There was a generalised consciousness on the need to keep snakes away from homes/compounds and to prevent snakebites, however, implementation was greatly limited by financial constraints. Healthcare facilities were generally accepted as the best place to seek healthcare following a bite (except for members of some religious groups that believed only in the power of prayers), and much preferable to consulting a traditional healer. However, poor road networks, limited ambulance services and transport options and lack of money all delayed or prevented victims from getting to hospitals. Almost everyone recognised the value of first aid and there was a specific appreciation of the utility of the black stone. Some religious groups believed only in the power of prayers. The removal of the “teeth” was an expected treatment outcome for some community members, failure of which explained poor wound healing or persistence of symptoms.

#### 4.1.1 Perception of snakes could inform outcomes of human-snake interactions

The negative perception of snakes in our study confirms findings from previous studies [7,25]. Snakes have been known to evoke ambivalent emotions in humans, ranging from a mixture of fear and reverence on one end, to disgust and ophidiophobia at the other end [26–28]. These perceptions are mostly born out of misconceptions, misunderstandings, and myths, which can be perpetuated and reinforced by a high frequency of snakes and a high incidence of snakebites. Snakes do not attack humans unprovoked, and they fear humans more than we fear them [29,30]. Low literacy levels and limited snake-specific knowledge in most rural areas in sub-Saharan Africa make it hard to challenge these misconceptions and change human behaviour towards snakes [7,31]. Furthermore, none of our participants mentioned a beneficial use of snakes. This is contrary to findings of snakes being used for food, medicine and magico-religious purposes among native populations in Mexico [25,32,33]. Also, the antihypertensive drug, captopril was first synthetically developed from the venom of *Bothrops jararaca*, and this medication is widely used in Kenya [34, 35].

Some of our participants held strong religious beliefs. They perceived snakebite as a biblical prescription and believed that only prayers should be used to prevent or treat snakebites. We did not collect data on the religious denominations of these participants. However, their constant referral to God and to Jesus Christ suggests a Christian denomination. These beliefs are similar to those shared by the Kavonokya sect, which has existed in the Ukambani and Kitui regions since 1914, and whose fundamental belief is that only God cures, and ‘worldly’ medicine and education should be shunned [36]. There have been confrontations with health officials in Kitui, mostly around the refusal by parents belonging to this sect to have their children vaccinated, and the reported deaths of hundreds of patients in rooms for ‘healing prayers’. [36]. Anecdotal reports suggest that snakebite victims are usually kept in these healing rooms. Conventional approaches to snakebite prevention and management are likely to fail in members of this group, hence the need for targeted interventions at the intersection of modern medicine and religion.

Relating other studies to our findings in communities in Kitui county portrays the heterogeneity in beliefs and perceptions of snakes, and snakebites among different communities within a country. Thus, Luo communities in western Kenya (550 km away from Kitui) regard some snakes as important fertility and harvest symbols, and this modifies their attitudes towards these snakes [37] in considerable contrast to our findings in Kitui communities. In communities which predominantly rely on agriculture, snakes could play a role in maintaining a stable ecosystem [38], and help improve agricultural productivity by controlling rodent populations consuming seed/grain harvests. There is evidence to suggest that these perceptions can be modified by learning more about snakes, and being in contact with harmless snakes with the support of herpetologists, for example in the case of rural Kenyan teachers whose conceptions of snakes became more favourable after attending a 6-hour seminar on reptiles, witnessing positive modelling of snake handling, and direct engagement with live harmless snakes [7,31]. The use of art forms such as the *Iculo ngenyoka* song from Eswatini, or the *Danza de los Negritos* cultural celebration in Mexico are innovative ways of learning about snakes that could appeal to a variety of audiences and result in generational transmission of knowledge [25,39]. We therefore recommend the following community outreach activities be explored to improve perceptions of snakes in Kitui county and other snake-affected rural communities: a) Research to identify community beliefs and identify misconceptions about snakes, and interventions to clarify and rectify them using locally developed knowledge and expertise; b) Using art forms to encourage learning about snakes in communities; c) Highlighting the positive uses of snakes for the ecosystem, agricultural productivity, medicine production, and other utilitarian purposes; and d) Organising snake handling workshops with trained herpetologists imparting the above messages in a memorable format.

#### 4.1.2 Context-specific education on prevention of snakebites

Community engagement to prevent snakebites is one of the four pillars of the WHO’s strategy to halve snakebite deaths and disability by 2030 [12]. Despite the varying perceptions and beliefs on the causes of snakebites, participants were knowledgeable on methods to keep snakes away and to prevent snakebites. Some interventions such as cleaning surroundings, using torches at nights, and wearing closed shoes could be positively reinforced and encouraged. A third of the participants in this study attested to have attended community education sessions organised by K-SRIC, and these measures are amongst those recommended by the Kenya Ministry of Health (MOH) and the World Health Organisation [WHO] [40,41]. The positive will to implement most of these measures was curbed by limited financial means, highlighting the need to suggest affordable and pragmatic preventive interventions, especially in poor communities. Some participants suggested other preventive measures such as burning trees and rubber and using paraffin to drive snakes away. Further research is needed to assess the snake-deterring effectiveness of these preventive measures, their ecological impact and to assess risks of burn and smoke inhalation injuries likely associated with these practices. In a Facebook post, the Kenya Wildlife Service (KWS) recommended eight home remedies to keep snakes away, some of which were: “3. Use natural predators; cats, chickens, ducks and dogs are known to keep snakes away”, “4. Smoke them away by burning any material with powerful fumes like plastic products”, and “5. Utilise products that work as snake repellents like kerosene, used engine oil by pouring them around the compound” [42]. Some of these remedies differ from those recommended by the Kenya MOH and the WHO and raises the need for coherence in community education on how to prevent snakes and snakebite [40,41]. This highlights the need for increased and coordinated multisectoral approach, through a national action plan, in reducing the burden of snakebite in Kenya.

#### 4.1.3 Involving victims in their management and meeting their expectations of healing

Almost all participants expressed a desire to seek formal healthcare in a hospital following a snakebite. This is contrary to previous studies in Kenya and elsewhere which reported patients preferring traditional healers to hospital care [43–45]. Our findings may reflect a recent behaviour change following an increase in community education activities promoting healthcare seeking behaviours, it may also identify a nuance, which is that people prefer visiting health facilities—provided they are affordable and can easily be assessed. The finding that communities in Kitui county prefer hospital-based treatment over consulting traditional healers is important as it underlines the importance of public health messaging, that is appropriately and sensitively delivered, in engendering behaviour changes in health seeking behaviours. When supported by well-equipped and affordable medical facilities, such messaging could significantly improve snakebite outcomes in endemic communities. Other challenges such as inaccessible health facilities, lack of available transport, and high cost of treatment may prevent patients from acting on their preferred health seeking behaviour [4,46]. Participants in our study identified logistic and financial obstacles to early hospital presentation following a snakebite. Suggesting that effective ambulance services and health financing schemes would improve the health seeking behaviours of snakebite victims. These findings can inform health policy formulation and identify key investment areas to improve health seeking behaviour.

Early interventions were considered by most participants as a temporary solution and/or a complimentary intervention to hospital treatment. The black stone was used in different ways and with different intended outcomes. All early interventions aimed to limit the spread of, or to get rid of the snake venom by sucking it out, or by causing the victim to vomit. There was some rationality to justify the use of these methods by most participants, suggesting a historical process of conception and rationalisation of these measures, often reinforced by varying degrees of success, probably due to the low proportion of snakebites that result in true envenoming–‘dry’ bites when venom is not injected - and bites from non-venomous snakes [37,45,47]. Interventions to discourage the use of these unproven first aid methods need to adopt a reverse process of deconstruction and de-rationalisation, using locally appropriate language and incorporating local beliefs and cultures. This echoes the need for more participatory and ethno-cultural methods in snakebite research [13,14]. A change in the epistemological conception of community education material is warranted, with a shift towards enhancing the value and use of local and traditional experience and knowledge.

The wide adoption of early intervention measures by participants in this study and the desire to access hospital care reinforces the need for effective close-to-community treatments which can be kept at the local dispensary, even at home and used immediately following a snakebite. In line with next generation snakebite treatments research on toxin-neutralising small molecules whose anticipated clinical efficacy, safety, affordability, non-cold chain dependence and oral delivery characteristics align with a community-dispensable snakebite treatment [48]. This would enable a transition from passive snakebite victims into active first line respondents, permitting snakebite victims to play an active and important role in improving outcomes following a snakebite.

We also found that participants reported having expectations, after seeking healthcare in a hospital, to return to their pre-bite state of health. An indicator of complete healing was when the snake’s fangs (teeth) fell out of their skin or of the wound, failure of which would result in persistent pain, delayed wound healing and other complications. This is a well-known misconception of snakes and snakebite and is wide spread in sub-Saharan Africa, stemming from the perception that all snakes are front-fanged and that a failure by a layperson to identify the fangs of a snake after it has been killed, is because the fangs broke off and are lodged in the victim’s skin or wound [27]. This specific misconception, alongside 19 others, have been researched and clarified in Northern Ghana [27]. There is a need to seek out other common misconceptions from other high burden areas, and to create context-appropriate community and patient education material to clarify them. Patient expectations should be appropriately managed by discussing possible outcomes, incorporating follow-up visits and addressing long-term complications of snakebites.

#### 4.1.4 Strengths and limitations

Our study benefited from a rigorous design, conduct and analysis. We used appropriate sampling and data collection techniques, and participants were recruited until data saturation was attained. The design and conduct received significant input from local researchers and public health authorities, resulting in highly authentic data. A systematic and iterative approach to data analysis ensured the trustworthiness of our analysis.

However, the interpretation of our findings in Kitui county should consider the following limitations. We used only semi-structured interviews to collect data, and an inability to triangulate our findings with other data sources may have reduced the quality of our data. The interpreter may have modified or summarised the responses of participants with consequences on the quality of our data.

## 5.0 Conclusion

In Kitui county, snakes were frequently encountered around households, they were generally perceived as dangerous and harmful animals which should be killed on sight, and only a handful of participants felt snakes would run away if given the chance. There is a need to explore and clarify common misconceptions about snakes, encourage learning about the true nature of snakes, and highlight the beneficial uses of snakes. There is a need for community outreach programs to promote the use of clinically proven first aid measures to replace unhelpful local early interventions and to reinforce the value of rapid access to hospital care. A change in the epistemological conception of community education material, by enhancing the value and use of local knowledge, and the employment of art techniques to transmit this knowledge, could improve community perception and methods of snakebite prevention. Participants identified logistic and financial obstacles to early hospital presentation following a snakebite. Suggesting that effective ambulance services and health financing schemes would improve the health seeking behaviours of snakebite victims. Religious belief appeared to be an important basis underpinning decisions on health seeking behaviours, both for and against seeking hospital care–and clearly needs more research. Finally, patients had expected treatment outcomes which were sometimes influenced by local misconceptions of snake anatomy. There is a need to seek out common misconceptions from other high burden areas, and to create context-appropriate community and patient education material to clarify these misconceptions. Patient expectations should be appropriately managed by discussing possible outcomes, incorporating follow-up visits and addressing long-term complications of snakebites.

## Supporting information

S1 Table Topic guide for interviews. (DOCX)

S2 Table COREQ checklist. (DOCX)
