# Supplementary material for: “Then they prayed, they did nothing else, they just prayed for the boy and he was well”: A qualitative investigation into the perceptions and behaviours surrounding snakebite and its management in rural communities of Kitui county, Kenya
Source: PLoS Negl Trop Dis. 2022 Jul 6;16(7):e0010579. doi: 10.1371/journal.pntd.0010579 (PMC9307190; doi:10.1371/journal.pntd.0010579)
Supplement: S1 Table — (DOCX) [file pntd.0010579.s001.docx]

***Appendix X*** *– Final topic guide*

| **Snakes - general** | What do you think of snakes? |
| --- | --- |
|  | Do you think there are lots of snakes around here? |
|  | Have you seen any snakes? / How often do you see snakes? |
|  | In what location do you most often see snakes? |
|  | What do you do if you see a snake? Why do you do that? |
| **Snakebite - general** | Have you, or anyone you know, been bitten by a snake? |
|  | What, do you believe, is the reason people are bitten by snakes? |
|  | Are all snakes poisonous? Do you know which ones are more poisonous? |
|  | Where do most snakebites occur on a person? |
|  | In what location do snakebites mostly occur? |
|  | Who is most at risk of being bitten by a snake? |
|  | Is there a time of day that snakebites are more likely to occur? |
| **Snakebite - protective measures** | Do you do anything to keep snakes away from the house or compound? |
|  | Do you do anything to prevent yourself from getting bitten? |
|  | What else could you do to protect yourself from snakebites? |
|  | Do you own closed shoes? Do you wear them/would you wear them if you did? |
| **Snakebite – actions following snakebite** | What would you do if you, or someone you knew were bitten by a snake? |
|  | Would you manage any snakebites at home? If so, how? |
|  | Have you or anyone you know managed a snakebite at home? If so, how? |
|  | Would you consider attending a traditional healer? |
|  | How would you decide what to do following a snakebite? What influences this? |
|  | Are there any reasons you would not attend hospital following a snakebite? |
|  | How long would you wait before seeking treatment? Do you think time is important? |
| **Additional** | Is there anything you would like to add or say? |
|  | Do you have any questions you would like to ask? |

*For interviewees with experience of snakebite:*

|  | What were you/they doing when they were bitten? |
| --- | --- |
|  | Do you know what snake it was? |
|  | What happened to you when you/they were bitten by a snake? |
|  | What did you/they do after being bitten? Management at home? |
|  | Did you/they receive any traditional treatments? |
|  | Did you/they go to a health care facility? |
|  | What influenced you/them to manage the bite in this way? |
|  | How long did it take to get to the health care facility? |
|  | How long was it before you/they decided to go to a health care facility? |
|  | Why did you/they delay this? |
|  | What treatment did you/they receive when they got to hospital? |
|  | How long did you/they stay in hospital? |
|  | How much did it cost? |
|  | What was the outcome? Have you/they had any long-term problems/complications from this bite? Economic/employment? Psychological? |
|  | How has this experience influenced how you would deal with future snakebites? |
|  | Have you taken any measures to prevent future snakebites? |
