# Supplementary material for: “Then they prayed, they did nothing else, they just prayed for the boy and he was well”: A qualitative investigation into the perceptions and behaviours surrounding snakebite and its management in rural communities of Kitui county, Kenya
Source: PLoS Negl Trop Dis. 2022 Jul 6;16(7):e0010579. doi: 10.1371/journal.pntd.0010579 (PMC9307190; doi:10.1371/journal.pntd.0010579)
Supplement: S2 Table — (DOCX) [file pntd.0010579.s002.docx]

**Consolidated criteria for reporting qualitative studies (COREQ): 32-item checklist**

| **No.** | **Item** | **Description** | **Section #** |
| --- | --- | --- | --- |
| **Domain 1: Research team and reflexivity** | | |  |
| Personal characteristics | | |  |
| *1.* | Interviewer/facilitator | Which author/s conducted the interview or focus group? | 2.4 |
| *2.* | Credentials | What were the researcher's credentials? *E.g. PhD, MD* | 2.4 |
| *3.* | Occupation | What was their occupation at the time of the study? | 2.4 |
| *4.* | Gender | Was the researcher male or female? | 2.6 |
| *5.* | Experience and training | What experience or training did the researcher have? | 2.5 |
| Relationship with participants | | |  |
| *6.* | Relationship established | Was a relationship established prior to study commencement? | 2.5 |
| *7.* | Participant knowledge of the interviewer | What did the participants know about the researcher? *E.g. Personal goals, reasons for doing the research* | 2.7 |
| *8.* | Interviewer characteristics | What characteristics were reported about the interviewer/facilitator? *E.g. Bias, assumptions, reasons and interests in the research topic* | 2.7 |
| **Domain 2: Study design** | | |  |
| Theoretical framework | | |  |
| *9.* | Methodological orientation and theory | What methodological orientation was stated to underpin the study? *E.g. grounded theory, discourse analysis, ethnography, phenomenology, content analysis* | 2.6 |
| Participant selection | | |  |
| *10.* | Sampling | How were participants selected? *E.g. purposive, convenience, consecutive, snowball* | 2.4 |
| *11.* | Method of approach | How were participants approached? *E.g. face-to-face, telephone, mail, email* | 2.5 |
| *12.* | Sample size | How many participants were in the study? | 2.4 |
| *13.* | Non-participation | How many people refused to participate or dropped out? What were the reasons for this? | - |
| Setting | | |  |
| *14.* | Setting of data collection | Where was the data collected? *E.g. home, clinic, workplace* | 2.5 |
| *15.* | Presence of non-participants | Was anyone else present besides the participants and researchers? | 2.5 |

|  |  |  |  |
| --- | --- | --- | --- |
| *16.* | Description of sample | What are the important characteristics of the sample? *E.g. demographic data, date* | 3.0 |
| Data collection | | |  |
| *17.* | Interview guide | Were questions, prompts, guides provided by the authors? Was it pilot tested? | 2.5 |
| *18.* | Repeat interviews | Were repeat interviews carried out? If yes, how many? | - |
| *19.* | Audio/visual recording | Did the research use audio or visual recording to collect the data? | 2.6 |
| *20.* | Field notes | Were field notes made during and/or after the interview or focus group? | 2.7 |
| *21.* | Duration | What was the duration of the interviews or focus group? | 2.5 |
| *22.* | Data saturation | Was data saturation discussed? | 2.4 |
| *23.* | Transcripts returned | Were transcripts returned to participants for comment and/or correction? | - |
| **Domain 3: analysis and findings** | | |  |
| Data analysis | | |  |
| *24.* | Number of data coders | How many data coders coded the data? | 2.6 |
| *25.* | Description of the coding tree | Did authors provide a description of the coding tree? | 3 |
| *26.* | Derivation of themes | Were themes identified in advance or derived from the data? | 2.6 |
| *27.* | Software | What software, if applicable, was used to manage the data? | 2.6 |
| *28.* | Participant checking | Did participants provide feedback on the findings? | - |
| Reporting | | |  |
| *29.* | Quotations presented | Were participant quotations presented to illustrate the themes / findings? Was each quotation identified? *E.g. Participant number* | 3 |
| *30.* | Data and findings consistent | Was there consistency between the data presented and the findings? | 3 |
| *31.* | Clarity of major themes | Were major themes clearly presented in the findings? | 3 |
| *32.* | Clarity of minor themes | Is there a description of diverse cases or discussion of minor themes? | 3 |

**Reference**

Allison Tong, Peter Sainsbury, Jonathan Craig, Consolidated criteria for reporting qualitative research (COREQ): a 32-item checklist for interviews and focus groups, International Journal for Quality in Health Care, Volume 19, Issue 6, December 2007, Pages 349–357, https://doi.org/10.1093/intqhc/mzm042
